# Efficacy and safety of peroral endoscopic myotomy for esophageal diverticula

**DOI:** 10.1055/a-2071-6744

**Published:** 2023-05-26

**Authors:** Elise M. Wessels, Jeroen M. Schuitenmaker, Barbara A.J. Bastiaansen, Paul Fockens, Gwen M.C. Masclee, Albert J. Bredenoord

**Affiliations:** 1 Amsterdam University Medical Centres, Gastroenterology & Hepatology, Amsterdam, Netherlands; 2 Amsterdam University Medical Centres, Gastroenterology & Hepatology, Duivendrecht, Netherlands

## Abstract

**Background and study aims** Epiphrenic diverticula are rare and mainly occur in patients with underlying esophageal motility disorders. The current standard treatment is surgical diverticulectomy often combined by myotomy and is associated with significant adverse event (AE) rates. The aim of this study was to examine the efficacy and safety of peroral endoscopic myotomy in reducing esophageal symptoms in patients with esophageal diverticula.

**Patients and methods** We performed a retrospective cohort study including patients with an esophageal diverticulum who underwent POEM between October 2014 and December 2022. After informed consent, data were extracted from medical records and patients completed a survey by telephone. The primary outcome was treatment success, defined as Eckardt score below 4 with a minimal reduction of 2 points.

**Results** Seventeen patients (mean age 71 years, 41.2 % female) were included. Achalasia was confirmed in 13 patients (13 /17, 76.5 %), Jackhammer esophagus in two patients (2 /17, 11.8 %), diffuse esophageal spasm in one patient (1 /17, 5.9 %) and in one patient no esophageal motility disorder was found (1 /17, 5.9 %). Treatment success was 68.8 % and only one patient (6.3 %) underwent retreatment (pneumatic dilatation). Median Eckardt scores decreased from 7 to 1 after POEM (p < 0.001). Mean size of the diverticula decreased from 3.6 cm to 2.9 cm after POEM (p < 0.001). Clinical admission was one night for all patients. AEs occurred in two patients (11.8 %) which were classified as grade II and IIIa (AGREE classification).

**Conclusions** POEM is effective and safe to treat patients with esophageal diverticula and an underlying esophageal motility disorder.

**Abbreviations**
GERD: gastroesophageal reflux disease
LES: lower esophageal sphincter
LHM: laparoscopic Heller’s myotomy
POEM: peroral endoscopic myotomy
D-POEM: diverticular peroral endoscopic myotomy

## Introduction

Most epiphrenic diverticula are pulsion diverticula and are associated with an underlying esophageal motility disorder 1 2 3 4 5 . It is estimated that 58 % to 78 % of patients with an epiphrenic diverticulum have an underlying esophageal motility disorder 1 2 3 . In patients diagnosed with achalasia, epiphrenic diverticula can occur due to stasis of food and increased intraluminal pressure in the distal esophagus 6 . This pressure results in herniation of the mucosal and submucosal layer, resulting in a so-called false diverticulum 7 . The prevalence of epiphrenic diverticula in achalasia ranges between 0.06 % to 4 % 8 . However, this may be an underestimation, as these estimates are derived from symptomatic achalasia patients in which the diverticulum is presumed to contribute to the symptoms. In the majority of cases, epiphrenic diverticula will remain asymptomatic 6 9 .

The current standard treatment of epiphrenic diverticula is surgical diverticulectomy often combined with laparoscopic Heller’s myotomy (LHM) and fundoplication. This procedure is successful in around 80 % of cases, but is rather invasive and carries a significant adverse event (AE) rate of 21 % to 27 % with an esophageal leakage rate of 7 % to 13 % 10 11 . Therefore, evolving surgical and endoscopic minimally invasive treatment approaches are increasingly considered. Peroral endoscopic myotomy (POEM) appears to be effective and safe for patients with achalasia and has mortality and morbidity rates of 0 % and 3.2 % to 7.5 % respectively 12 13 . Previous studies have suggested that symptoms in patients with an epiphrenic diverticulum are being caused by the underlying esophageal motility disorder rather than the epiphrenic diverticulum itself 14 . Given the fact that esophageal motility disorders are mainly the underlying cause of epiphrenic diverticula, the idea is to treat primarily the underlying cause by myotomy without diverticulotomy to reduce symptoms as well as the chance of developing new diverticula. In case of persistent symptoms after POEM, a second intervention can be considered for additional diverticulotomy, diverticulectomy or resection of the diverticular pouch 8 14 15 16 .

To date, only a small number of studies have investigated the efficacy and safety of POEM without diverticulotomy in patients with esophageal diverticula and they are limited by the number of included patients 17 18 19 20 21 22 23 24 25 26 . The largest study thus far with 14 patients showed a decrease in Eckardt score after POEM. In total 20 patients have been reported in case series and case reports and symptoms improved in all of them after POEM. The aim of this study was to examine the efficacy and safety of POEM without diverticulotomy in reducing esophageal symptoms in patients with esophageal diverticula.

## Patients and methods

### Study design

This retrospective cohort study was performed at the Amsterdam University Medical Center. Data were collected from patients with an esophageal diverticulum who underwent POEM between October 2014 and December 2021. Baseline characteristics and follow-up variables were extracted from medical records. Patients who were included in the study were contacted by telephone to complete a survey (appendix). Patients consented to the use of their medical data for the purpose of this study.

### Patient selection

Medical records of all consecutive patients who underwent POEM were screened for eligibility. Barium esophagograms were used to confirm the esophageal diverticula. Inclusion criteria were one or more esophageal diverticula diagnosed before POEM and an age > 18 years. Also, POEM had to be carried out successfully and the diverticula had to be documented during the procedure or during a previous gastroscopy. Patients with previous or current malignancy of the esophagus, gastroesophageal surgery in the past or a Zenker diverticulum were excluded.

### POEM procedure

All POEM procedures were performed under general anesthesia with endotracheal intubation and patients received perioperative intravenous antibiotics. The procedure was carried out by two gastroenterologists with significant POEM experience (PF, BB) according to the steps as follows: (1) a submucosal injection of saline and indigo carmine followed by a mucosal incision of 2 cm approximately halfway down the esophageal body; (2) creation of a submucosal tunnel up to the cardia to approximately 3 cm beyond the LES; (3) myotomy of the circular muscular layer as well as part of the longitudinal muscular layer; and (4) closure of the mucosal incision with multiple endoclips. The length of the myotomy depended on the location of the diverticula and the type of underlying esophageal motility disorder. Myotomy of the diverticular septum was not performed.

### Barium esophagogram

A timed barium esophagogram was performed before POEM and between 3 and 18 months after POEM. The patient had to swallow 200 mL of barium contrast within 15 to 20 seconds while upright. Radiographs of the esophagus were made at baseline and after 1, 2 and 5 minutes 20 . The maximum size of the diverticula was measured in centimeters. In some patients, more than one barium esophagogram was performed at different times of follow up. The mean maximum size of the diverticula was used in case of multiple barium esophagograms after POEM because in one patient, the maximum size of the diverticula on repeated barium esophagogram after POEM did not differ significantly. The size of the diverticula was measured independently by two observers. The mean size was used when the difference between the values measured by the observers was < 0.5 cm. Otherwise, the size was measured again until agreement was reached.

### Outcome measures

The primary outcome was treatment success, defined as an Eckardt score < 4 with a minimum decrease of 2 points after POEM 27 . The Eckardt score was used to quantify the esophageal symptoms and was calculated just before POEM and at the time of the survey. Symptoms of weight loss, dysphagia, retrosternal pain and regurgitation were scored from 0 to 3 indicating the severity of the symptoms, resulting in a total maximum score of 12. The higher the score, the higher the burden of symptoms. Secondary outcomes included retreatment after POEM, self-reported improvement of esophageal complaints measured on a 6-point Likert scale, symptoms of gastroesophageal reflux disease (GERD symptoms), reflux esophagitis observed during gastroscopy 3 months after POEM, proton pump inhibitor (PPI) use and difference in size of the diverticula at follow up. Some patients had more than one diverticulum and these diverticula were analyzed separately. Therefore, the number of diverticula was higher than the number of patients in this study. Secondary outcomes related to the procedure were length and location of myotomy, number of days in hospital, procedure-related events and AEs within 30 days after POEM.

### Statistical analysis

Castor EDC was used for data management and all statistical analyses were performed using SPSS version 26.0. The distribution of variables was assessed by plotting a histogram and a quantile-quantile plot. Normally distributed outcomes were analyzed using paired sample *t* -test. Non-parametric testing (the Sign test and Wilcoxon test) was used to compare not normally distributed outcomes. *P* < 0.05 was considered to be statistically significant.

## Results

### Patient characteristics

**Fig. 1:**
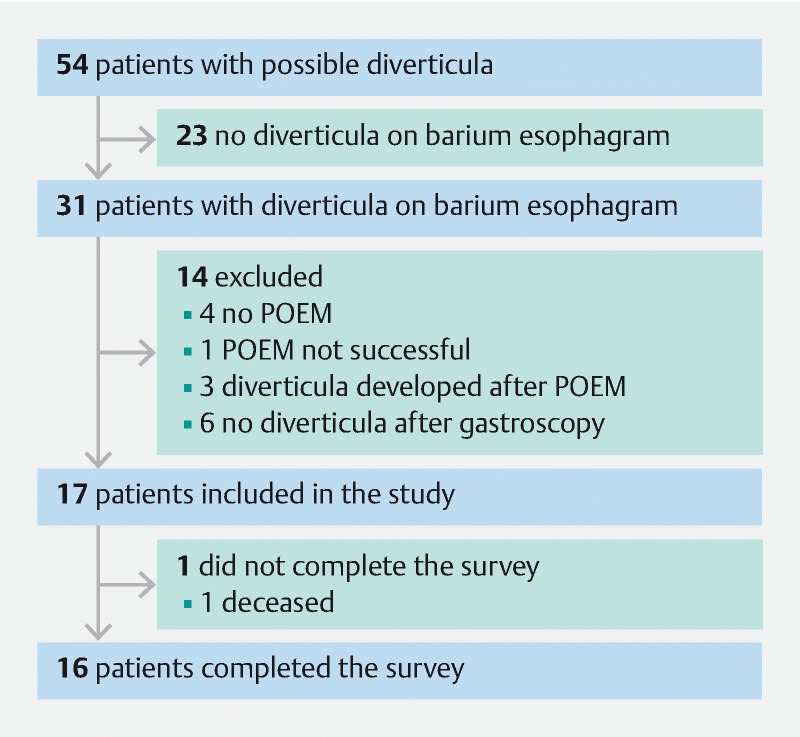
Flowchart. POEM, peroral endoscopic myotomy.

After screening lists of 220 patients who underwent POEM and 327 patients with achalasia, the charts of 54 patients mentioned possible presence of esophageal diverticula based on reports of diagnostic tests and procedures or referral letters. Diverticula were not confirmed on barium esophagogram in 23 of these patients. Of the 31 patients with confirmed diverticula, 14 were excluded because they did not meet other eligibility criteria. In six of the 14 patients, the diverticulum was not confirmed during gastroscopy, four patients did not undergo POEM, one POEM was not successful, and in three patients, the diverticulum was diagnosed after POEM. Of 17 included patients, one died for whom no data were obtained on the survey. The cause of death was not related to the procedure or the esophageal condition ( Fig. 1 ).

The variables age, body mass index and the maximum size of the diverticula were normally distributed. As shown in Table 1 , the mean age was 71 years and 41.2 % of the patients were female. Achalasia was confirmed in 13 patients (76.5 %), Jackhammer esophagus in two patients (11.8 %), diffuse esophageal spasm in one patient (5.9 %) and one patient showed no evidence of an esophageal motility disorder (5.9 %). About one-third of the patients underwent pneumatic dilatation in the past (6/17, 35.3 %) of whom three also underwent a LHM (17.6 %). The only patient without an esophageal motility disorder did have a surgical thoracoscopic diverticulectomy in the past and one patient was treated with nifedipine and isosorbide dinitrate because of diffuse esophageal spasm. Seven patients did not have any treatment before POEM (41.2 %). Most patients had one diverticulum (13/17, 76.5 %). Three patients had two diverticula and one patient had three. Of all these diverticula, 21 were epiphrenic and one was located in the lower part of the mid-esophagus. The size of the diverticula ranged from 0.9 cm to 8.3 cm with a mean of 3.7 cm.

**Table 1:** Baseline characteristics.

Patient characteristics	N = 17
Age, years, mean (SD)	71 (9)
Sex	
Female	7 (41.2 %)
Male	10 (58.8 %)
BMI, kg/m ^2^ , mean (SD)	25.3 (4)
EMD 1	
Achalasia	13 (76.5)
Type I	3 (17.6)
Type II	6 (35.3)
Type III	2 (11.8)
Jackhammer esophagus	2 (11.8)
DES	1 (5.9)
Normal	1 (5.9)
Time between EMD diagnosis and POEM, months, median (IQR)	10.5 (28.8)
Previous treatment	
PD	3 (17.6)
BTI	2 (11.8)
PD and LHM	2 (11.8)
PD and LHM and BTI	1 (5.9)
Surgical diverticulectomy	1 (5.9)
Other	1 (5.9)
None	7 (41.2)
Number of diverticula	
1	13 (76.5)
2	3 (17.6)
3	1 (5.9)
Diverticulum characteristics	N = 22
Location	
Mid-esophageal	1 (4.5)
Epiphrenic	21 (95.5)
Maximum size, cm, mean (SD)	3.9 (1.8)
Side	
Left	9 (40.9)
Right	13 (59.1)

Results are presented as n (%) unless otherwise stated.

SD, standard deviation; BMI, body mass index; EMD, esophageal motility disorder; DES, diffuse esophageal spasm; POEM, peroral endoscopic myotomy; IQR, interquartile range; PD, pneumatic dilatation; BTI, botulinum toxin injection; LHM, laparoscopic Heller’s myotomy.

1 Based on Chicago classification version 3.0.

### Treatment outcomes

**Fig. 2:**
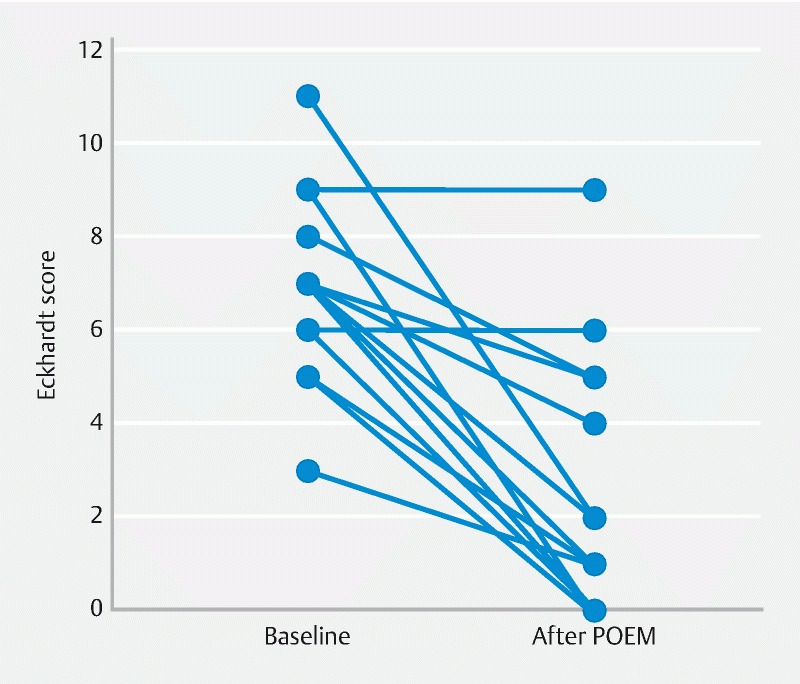
Eckardt scores before POEM and at follow up.

Treatment success was obtained in 68.8 % of patients. The median time between POEM and the survey was 31 months with a range from 6 to 93 months. Eckardt scores were not normally distributed. As shown in Table 2 , the Eckardt scores significantly decreased from a median of 7 (range 2–11) before POEM to a median of 1 (range 0–9) after POEM ( *P* < 0.001). From the graph in Fig. 2 , it can be seen that two patients had no difference in Eckardt score and the other 14 patients had a considerable decrease, of whom five patients had an Eckardt score of 0 and four patients a score of 1 after POEM. A decrease in Eckardt symptom score is seen for each symptom separately and is shown in **Supplementary 1** . Only one patient (6.3 %) had received retreatment after POEM, which was pneumatic dilatation.

**Table 2:** Treatment outcomes.

	Before POEM	After POEM	*P* value
Eckardt score, median (IQR) 1	7 (3)	1 (5)	< 0.001
Dysphagia	3 (0)	1 (2)	
Weight loss	0 (2)	0 (0)	
Retrosternal pain	1 (3)	0 (1)	
Regurgitation	2 (2)	1 (1)	
Maximum size diverticula, cm, mean (SD) 2	3.6 (0.4)	2.9 (0.3)	< 0.001
Self-reported improvement 1			
No complaints		7 (43.8)	
Marked improvement		3 (18.8)	
Some improvement		3 (18.8)	
No appreciable change		0 (0)	
Worsening		1 (6.3)	
Recurrence of symptoms after initial improvement		2 (12.5)	
Reflux esophagitis 1		7 (43.8)	
Grade A		3 (18.8)	
Grade B		2 (12.5)	
Grade C		1 (6.3)	
Grade D		1 (6.3)	
PPI use 1		12 (75)	
Retreatment 1		1 (6.3)	
PD		1 (6.3)	

Results are presented as n (%) unless otherwise stated.

IQR, interquartile range; PD, pneumatic dilatation; POEM, peroral endoscopic myotomy; SD, standard deviation; PPI, proton pump inhibitor.

1 n = 16.

2 n = 21.

**Fig. 3:**
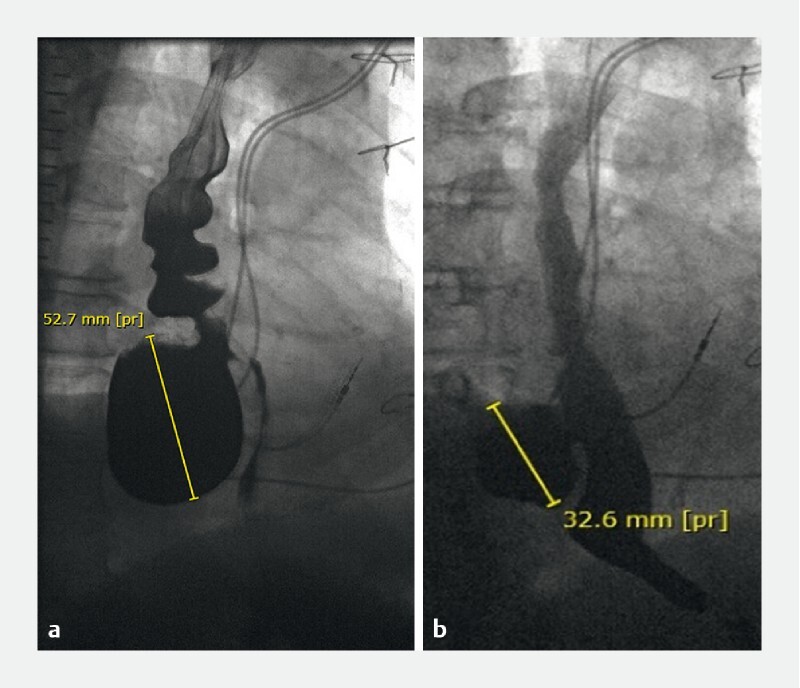
Barium esophagogram of a patient with a large epiphrenic diverticulum. **a** before POEM. **b** after POEM.

Four patients had more than one diverticulum and only one patient had no barium esophagogram on follow up, so the difference in maximum size of the diverticula was analyzed for 21 diverticula. As can be seen in Table 2 , the mean maximum size of the diverticula significantly decreased from 3.6 cm before POEM to 2.9 cm after POEM (mean difference 0.76 cm; 95 % CI 0.45–1.07; *P* < 0.001). Fig. 3 shows an example of the effect of POEM on a large diverticulum. Table 2 also provides the results of the self-reported improvement of esophageal complaints after POEM. In total 13 patients noticed improvement, seven of whom had no complaints. Of 16 patients who underwent gastroscopy after POEM, seven patients (41.2 %) had reflux esophagitis, in whom three were grade A, two grade B, one grade C and one grade D. Besides, GERD symptoms were reported as “sometimes” in 50 % and “often” in 6.3 % of patients. PPIs, mainly omeprazole, were used by 75 % of the patients. Four patients used PPIs once a day, seven patients twice a day and one patient only used a PPI when experiencing GERD symptoms. GERD symptoms were well controlled with PPIs in all patients.

### Procedure-related outcomes

Table 3 provides an overview of the procedure-related outcomes. Procedure time and the number of days in hospital were normally distributed. Total length of myotomy and the time between diagnosis of esophageal motility disorder and POEM did not follow a normal distribution. The median time between date of diagnosis of esophageal motility disorder and POEM was 10.5 months with a range from 0 to 53 months. The length of myotomy differed from 7 cm to 16 cm with a mean length of 10.5 cm. Clinical admission for all patients was one night. Mucosal injury occurred during one procedure, which was immediately noticed and closed with four extra hemoclips. This procedure-related event had no influence on patient outcome or hospital stay duration. Two AEs were reported within 30 days after POEM and were classified as grade II and grade IIIa according to the AGREE classification 28 .

**Table 3:** Procedure-related outcomes.

	N = 17
Total length of myotomy, cm, median (IQR)	10 (4)
Location of myotomy	
Next to diverticulum	12 (70.6)
Opposite of diverticulum	5 (29.4)
Procedure time, minutes, mean (SD)	88 (34)
Number of days in hospital, days, mean (SD)	2 (0)
Procedure-related events	1 (5.9)
Mucosal injury	1 (5.9)
Adverse events < 30 days after POEM 1	2 (11.8)
Grade II	1 (5.9)
Grade IIIa	1 (5.9)

Results are presented as n (%) unless otherwise stated.

1 Based on Classification for Adverse events Gastrointestinal Endoscopy (AGREE). IQR, interquartile range; SD, standard deviation; POEM, peroral endoscopic myotomy.

## Discussion

The results of this study suggest that POEM is an effective and safe treatment for patients with an esophageal diverticulum. Of the 17 patients included, 16 had an underlying esophageal motility disorder showing a large overall reduction in symptoms as well as a reduction in diverticulum size. Only one patient required retreatment after POEM and in the other 16 patients, the effect of POEM was sufficient that additional treatment was deemed unnecessary. One patient had normal esophageal motility and did not benefit from the POEM treatment. Thus, it seems imperative to carefully consider whether an esophageal diverticulum in a patient without underlying esophageal motility disorder is truly the cause of the symptoms.

There is a small number of series describing the effect of POEM without diverticulotomy for esophageal diverticula. A retrospective study by Kinoshita et al (2020) concluded that POEM alone for patients with an esophageal motility disorder and an epiphrenic diverticulum was effective and safe. In their study with 14 patients, the median Eckardt score significantly decreased from 5 (range 2–11) before POEM to 0 (range 0–2) after POEM. No difference in perioperative complications in patients with and without epiphrenic diverticula was observed 8 . Further, only small case series and case reports are published that concluded that POEM without diverticulotomy was safe and effective as treatment for patients with an esophageal diverticulum 17 18 19 20 21 22 23 24 25 26 .

In a randomized controlled trial in which the effect of POEM was compared to pneumatic dilatation in patients with achalasia, the treatment success rate of POEM was 92 % after 2 years 29 . When comparing the effect of POEM to LHM, the treatment success rates for POEM were 94.6 % and 83 % after 3 months and 2 years, respectively 30 . These treatment success rates differ from the findings in our current series. A possible explanation for this difference might be that our series consisted of a more heterogeneous group and only 77 % of the patients were diagnosed with achalasia.

POEM appeared to be safe for patients with esophageal diverticula and an underlying motility disorder. In total, one procedure-related event was reported, which had no influence on patient outcome or hospital stay duration. Two AEs within 30 days after POEM occurred in our patients. Both patients were readmitted because of postoperative retrosternal pain with need for non-opioid pain control in one patient and the other patient received a duodenal feeding tube for 25 days because of partial dehiscence of the mucosal incision that healed under conservative management.

Surgical diverticulectomy is successful in reducing symptoms of esophageal diverticula but carries a relatively high AE rate of 21 % to 27 % with suture leakage as the most frequently recorded complication with rates up to 13 % 10 11 . The potential benefit of an intervention for the esophageal diverticula must always be weighed against the complication risk. Zaninotto et al (2008) compared the outcomes of patients with esophageal diverticula after surgical diverticulectomy and after a conservative approach. They concluded that surgical diverticulectomy was effective in reducing esophageal symptoms. On the other hand, it was also safe to treat asymptomatic or mildly symptomatic patients conservatively 31 . To date, no study has been performed comparing the outcome of patients with esophageal diverticula undergoing POEM and a conservative approach. From our results, it seems that the size of the diverticulum is not related to complaints or complication risk, and therefore, it is questionable whether the size of the diverticulum should play a role in determining the indication for intervention 14 .

The main disadvantage of POEM is the risk of post-procedure gastroesophageal reflux. In this study, the GERD symptoms did not correspond with the presence of reflux esophagitis. Three of seven patients with reflux esophagitis had no GERD symptoms. In addition, six of nine patients without reflux esophagitis did have GERD symptoms. GERD symptoms were well controlled with PPIs, but the dose varied among patients. Previous studies reporting the risk of reflux esophagitis and GERD symptoms after POEM are not conclusive. Usually, GERD symptoms are less frequent after POEM than reflux esophagitis 32 . As compared to LHM, fundoplication is usually not performed during POEM, and therefore, the risk of reflux esophagitis is higher after POEM. However, transoral incisionless fundoplication is an emerging minimally invasive endoscopic fundoplication technique but the long-term efficacy and safety is unknown 33 . With longer follow up, the difference in prevalence of reflux esophagitis between POEM and LHM becomes smaller.

There are a few limitations of this study. Because esophageal diverticula are rare and POEM is not a standard intervention, the sample size was relatively small. Nonetheless, together with the study by Kinoshita et al (2020), this was the largest study evaluating the effect of POEM in patients with esophageal diverticula 8 . Another limitation is that the time between POEM and the survey varied and ranged from 6 to 93 months after POEM. From the survey, however, it can be concluded that there is a considerable improvement with a decrease in Eckardt scores in most patients, even after a long period of follow up. In view of the retrospective study design, no control group was included in this study. Treatment effect and AEs, therefore, could not be compared with standard care or other treatment.

POEM may reduce the resistance in the distal esophagus that makes food pass more easily, and thus, reduce intraluminal pressure. The underlying esophageal motility disorder is treated with POEM as well. This will contribute to reduction in symptoms and prevent development of new diverticula. When a patient has an esophageal diverticulum, a diverticular peroral endoscopic myotomy (D-POEM) can be performed in which septum division is carried out in addition to the esophageal myotomy 34 . This treatment appeared to be effective and safe for patients with esophageal diverticula, although samples sizes of reported studies are small 35 36 37 38 39 40 . Some authors suggest that D-POEM is preferred because septotomy is relatively easy to perform and additional diverticulotomy after a previous POEM can be difficult 37 . Our data suggest that additional diverticulotomy might not be necessary to achieve satisfactory treatment results. However, not all patients were asymptomatic after POEM and therefore, treatment can be further optimized. Persistent symptoms might be explained by the remaining diverticular pouch or sometimes the diverticulum may not be the cause of all symptoms after all. Further research is required to compare effect and safety of D-POEM versus POEM without diverticulotomy, to establish the long-term effects of POEM in patients with esophageal diverticula and to compare the effect with other treatments. Also, questions about the timing of intervention remain to be elucidated.

## Conclusions

In conclusion, our data provide further evidence that POEM is feasible, safe and effective as treatment for patients with esophageal diverticula and an underlying esophageal motility disorder.

### Contributorʼs statement

EMW, JMS and AJB designed the study. JMS and AJB supervised the project. EMW collected the data and was responsible for project administration. EMW performed the data and statistical analysis. All authors contributed to the interpretation of the results. EMW wrote the manuscript with input from all authors. All authors had full access to the data and approved the final manuscript.

## Supplementary material

Supplementary material Zusatzmaterial
