# Supplementary material for: Efficacy and safety of peroral endoscopic myotomy for esophageal diverticula
Source: Endosc Int Open. 2023 May 26;11(5):E546–52. doi: 10.1055/a-2071-6744 (PMC10219786; doi:10.1055/a-2071-6744)

Supplementary material

APPENDIX: Questionnaire after POEM

Procedure

1. Did any complication occur after POEM?
   - ☐ No
   - ☐ Yes, namely ________________________

After POEM

2. Did the esophageal symptoms change after POEM?
   - ☐ No complaints anymore
   - ☐ Marked improvement
   - ☐ Some improvement
   - ☐ No appreciable change
   - ☐ Worsening
   - ☐ Recurrence of symptoms after initial improvement

3. Did you underwent another treatment after POEM because of persistent of recurrent esophageal symptoms?
   - ☐ No
   - ☐ Yes, namely ________________________

Current symptoms

4. How often do you have difficulty with swallowing or the feeling that food is stuck in your esophagus?
   - ☐ None
   - ☐ Occasionally
   - ☐ Daily
   - ☐ Every meal

5. How often do you have regurgitation?
   - ☐ None
   - ☐ Occasionally
   - ☐ Daily
   - ☐ Every meal

6. How often do you have chest pain?
   - ☐ None
   - ☐ Occasionally
   - ☐ Daily
   - ☐ Every meal

7. Did you recently lost weight?
   - ☐ None
   - ☐ < 5 kg

Supplementary material

☐ 5-10 kg
☐ > 10 kg

8. Do you experience heartburn, burning chest pain or acid regurgitation?
☐ No
☐ Occasionally
☐ Often

9. Do you use any of the following acid-suppressive medication?
☐ Omeprazole
☐ Esomeprazole
☐ Pantoprazole
☐ Other, namely ___________________
☐ No acid-suppressive medication

10. Which dose and how many times a day do you use acid-suppressive medication?
______ mg _____ a day

11. When did you start with acid-suppressive medication?
☐ Before POEM
☐ Directly after POEM
☐ A while after POEM

12. Why did you start with acid-suppressive medication?
☐ Symptoms of heartburn, burning chest pain or acid regurgitation
☐ Because it was advised after POEM
☐ Because it was advised after standard upper endoscopy after POEM
☐ Because it was advised after 24-hour pH-impedance testing after POEM
☐ Other reason, namely ______________________

Supplementary material

Supplementary figure 1 Eckardt score for each symptom separately before POEM and at follow-up.

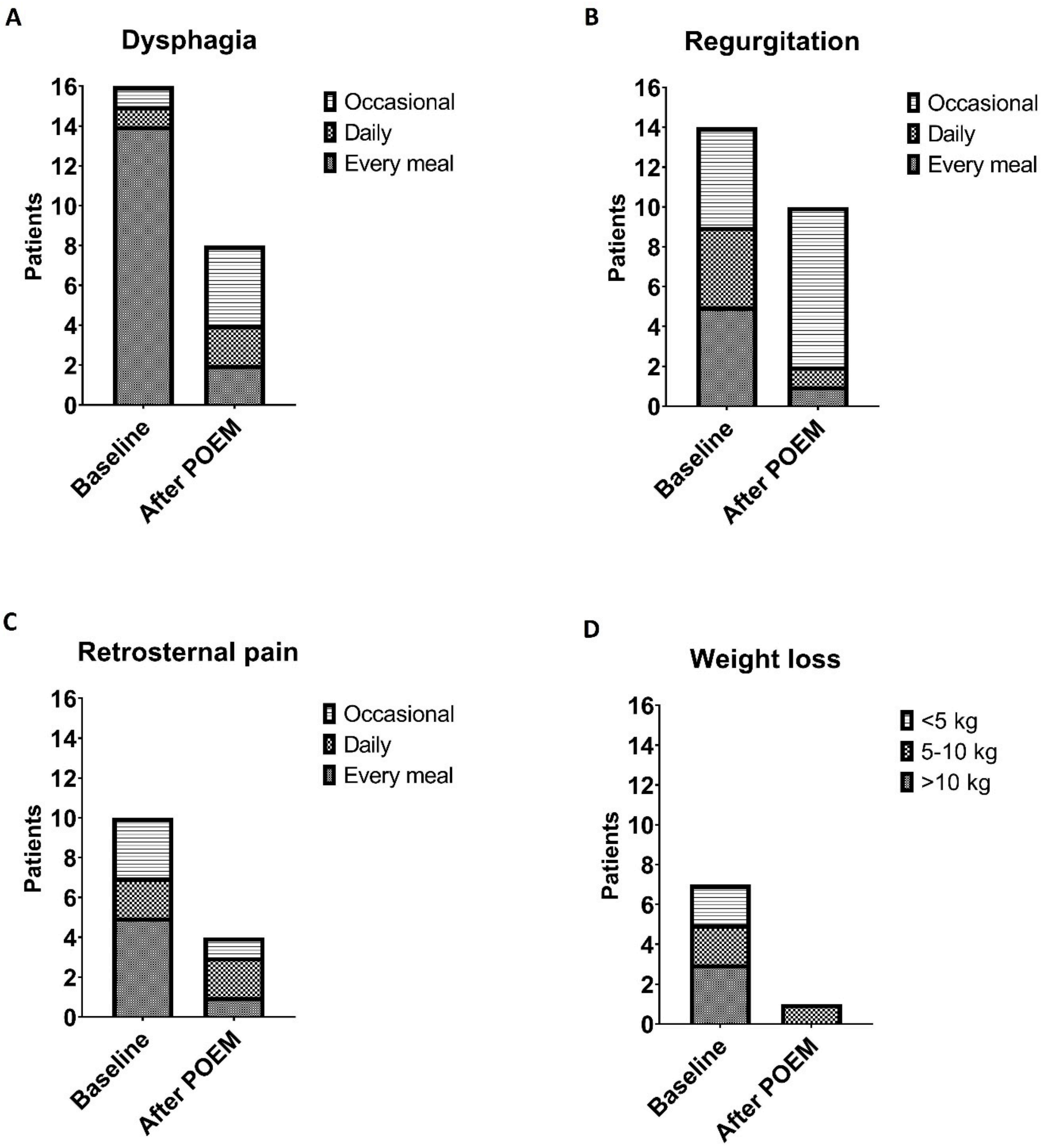

Supplement: Supplementary file 1 — Supplementary material [file 2946supmat_10-1055-a-2071-6744.pdf]
